# Supplementary material for: Aging and the Spectral Properties of Brain Hemodynamics
Source: Adv Sci (Weinh). 2025 Jul 16;12(37):e17644. doi: 10.1002/advs.202417644 (PMC12499389; doi:10.1002/advs.202417644)
Supplement: Supplementary file 1 — Supporting Information [file ADVS-12-e17644-s001.docx]

**Supplementary Materials**

**Summary of demographic characteristics for the two datasets used in this study**

|  | CamCAN (Cambridge Centre for Ageing and Neuroscience) | AMBR (Aging Metabolism & Brain Resilience) |
| --- | --- | --- |
| N | 455 | 94 |
| Age (yrs) | 18-87 | 25-45 (N = 30); 65-85 (N=64) |
| Sex (M:F) | 276:179 | 16:14; 13:21 |

**Analyzing BOLD fluctuations in the temporal frequency domain**

Prior analyses of BOLD signal fluctuations have largely focused on the amplitude of low-frequency fluctuations (ALFF) (Zang, He et al. 2007) and fractional ALFF (fALFF) (Zou, Zhu et al. 2008). Comparatively, fewer studies have focused on the spectral slope (He 2011, Baria, Mansour et al. 2013). ALFF quantitates the average power within a selected infra-slow frequency range (typically, <0.1 Hz). In contrast, fractional ALFF (fALFF) represents the relative prevalence of slow vs. fast activity. Accordingly, fALFF quantifies the proportion of infra-slow frequency activity relative to the full frequency spectrum (footnote: the range of measurable frequencies depends on the volume TR and fMRI run duration). Spectral slope characterizes the power spectrum in terms of a single number. This is possible because BOLD fluctuations are approximately scale-free, i.e., exhibit a 1/f-like power spectrum (Baria, Mansour et al. 2013, Tagliazucchi, von Wegner et al. 2013). Thus, the SS measure captures information similar to fALFF, although at a finer spectral resolution and utilizing more of the frequency spectrum.

**Log-log vs. log-linear spectral slope fitting**

Prior rs-fMRI work modeled BOLD signal spectral slope either as a log-log or log-linear relation (He 2011, Baria, Mansour et al. 2013, Park, Snyder et al. 2023). In the present study, we first compared the spectral slope fit using both approaches: a log-log analysis where both power and frequency were logarithmically transformed; and a log-linear analysis where only power was logarithmically transformed. For each method, a linear model described the relations between frequency and power. Specifically, the spectral slope was computed as the negative of the first derivative of the fitted slope.

To measure the model accuracy, we computed the coefficient of determination (R^2^), which computes the proportion of variance in the dependent variable predictable from the independent variable. This was calculated by first summing the squared deviations between the fitted slopes and the actual spectra to obtain the residual sum of squares, which indicates the variance unexplained by the model. The total sum of squares was computed by summing the squared deviations of the actual power spectra values from their mean, reflecting the total variance observed. R^2^ was derived by subtracting the ratio of residual sum of squares to the total sum of squares from one, with values closer to 1 indicating a more accurate model.

R^2^ values were consistently higher with the log-linear models, suggesting a better fit compared to the log-log approach (**Fig. S1**). Based on these findings, we used the log-linear slope fitting for our spectral slope metric.


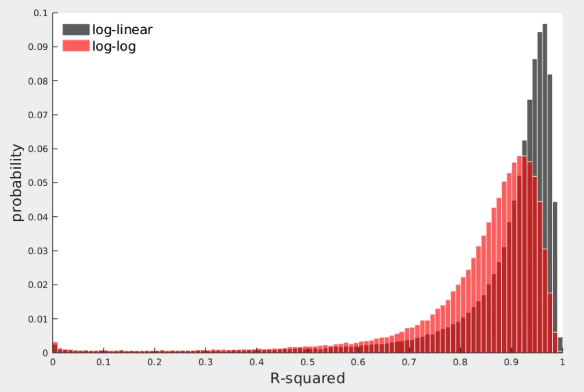


**Figure S1**. Distribution of R^2^ values of spectral slope fits in log-linear and log-log domains. The log-linear domain (gray) consistently shows higher R^2^ values compared to the log-log domain (red).

**Spin permutation test: rotation quaternions**

To test the significance of any model, it is useful to generate a null hypothesis. Here, the null hypothesis is no difference in the spatial similarity between the age effect in the spatial topography of spectral slope or CMRGlc. It is necessary to include spatial autocorrelation in the construction of the null model. Prior methodology to construct an appropriate null model, i.e., the spin test (Gordon, Laumann et al. 2016, Alexander-Bloch, Shou et al. 2018), generated surrogate data by randomly rotating brain surface distributions represented on the inflated sphere. In detail, the distribution of rotations achieved by this prior version of the spin test was not uniform. The present methodology achieves a uniform distribution of rotations by sampling random quaternions.

A quaternion is defined as four components: one real and three imaginary parts. These components are computed using the following formula:

$$q = [\sqrt{1-u1}\sin\left( 2\pi u2 \right),\sqrt{1-u1}\cos\left( 2\pi u2 \right),\sqrt{u1}\sin\left( 2\pi u3 \right),\sqrt{u1}\cos\left( 2\pi u3 \right) ]$$

where u1, u2, and u3 are uniformly distributed random numbers between 0 and 1. Note that $\sqrt{u1}\cos\left( 2\pi u3 \right)$ is the real part of the quaternion and the remaining components are the vector part of the quaternion. This formulation ensures a uniform distribution in the rotation group $SO(3)$.

To apply the rotation, each quaternion is converted into a corresponding 3x3 rotation matrix, R:

$$R= \left[ \begin{matrix} 1-{2q}_{y}^{2}-{2q}_{z}^{2} & {2q}_{x}q_{y}-{2q}_{z}q_{w} & {2q}_{x}q_{z}+{2q}_{y}q_{w} \\ {2q}_{x}q_{y}+{2q}_{z}q_{w} & 1-{2q}_{x}^{2}-{2q}_{z}^{2} & {2q}_{y}q_{z}-{2q}_{x}q_{w} \\ {2q}_{x}q_{z}-{2q}_{y}q_{w} & {2q}_{y}q_{z}+{2q}_{x}q_{w} & 1-{2q}_{x}^{2}-{2q}_{y}^{2} \end{matrix} \right]$$

The variables $q_{w}$, $q_{x}$, $q_{y}$, and $q_{z}$ represent the real and imaginary parts of quaternion $q$, respectively. This matrix applies the quaternion’s defined spatial orientation to rotate each vertex. (See (Kuipers 1999) Chapter 5, Section 5.14 “Quaternions to Matrices” for more details.)

The remaining operations are performed as described in (Gordon, Laumann et al. 2016, Alexander-Bloch, Shou et al. 2018). In brief, both the centroid vertices and the corresponding age-specific R^2^ difference values are reflected across the Y-Z plane. The quaternion used for the left hemisphere is also applied to the right hemisphere for each random sample rotation. Centroid vertices rotated into the medial wall are excluded when computing the Spearman’s correlation between the rotated values and CMRGlc values.

**Fuzzy Silhouette score analysis**

Trajectories in age-related spectral slope flattening were not uniform (main text **Fig. 2**). Accordingly, we performed a silhouette score analysis (Rousseeuw 1987) using the fuzzy c-means clustering algorithm to determine the optimal number of clusters. Fuzzy c-means clustering was applied to z-scored GAM smooth functions that chart the trajectory of SS changes with age, across a range of 2 to 15 clusters.

Silhouette score analysis requires hard cluster assignments. Accordingly, we first converted fuzzy membership scores to hard assignments by assigning the parcel to the cluster with the highest membership value. Subsequently, we computed the silhouette scores for each parcel using the Euclidean distance metric. The silhouette value of each parcel quantifies its similarity to other parcels within its own cluster compared to parcels in the nearest cluster. The average silhouette score for each cluster number was computed by averaging the silhouette scores across all parcels (**Fig. S2**). The final result showed that the optimal number of clusters was 2.

**
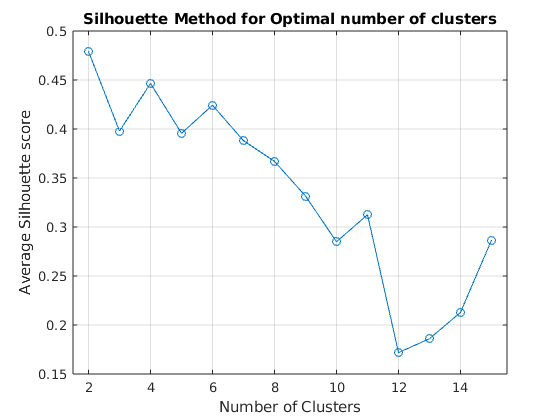
**

**Figure S2**. Average silhouette scores for each cluster number, ranging from 2 to 15. Higher silhouette scores indicate better cluster cohesion and separation.

**Linear regression model result**

**Supplementary Table 1.** Relation between outlier status and frontal lobe white matter intensity (p < 2e-06). Linear regression model (*fitlm* function in MATLAB 2023b) results including grey matter volume (GMvol), head motion (HM), sex, age, and T2-weighted frontal lobe white matter intensity as independent variables, and outlier status as the dependent categorical variable. See main text Figure 5. Note that only subjects older than 45 years is included in this analysis.

$$SS Youthful Index \sim\beta_{o}+\beta_{1}age+ \beta_{2}GMV+ \beta_{3}HM+ \beta_{4}sex+\beta_{5}FLWM T2w$$

|  | Estimate | Standard Error | tStat | p value |
| --- | --- | --- | --- | --- |
| (Intercept) | 2.7451 | 0.54374 | 5.0485 | 7.7805e-07 |
| GMvol | 0.3624 | 0.76227 | 0.47543 | 0.63483 |
| HM | -0.097598 | 0.14431 | -0.67632 | 0.49936 |
| sex | -0.052252 | 0.030673 | -1.7035 | 0.089515 |
| age | 0.00098343 | 0.0016031 | 0.61347 | 0.54004 |
| T2wWM | -7.399 | 1.5253 | -4.8509 | 1.9865e-06 |

**Supplementary Table 2.** Relation between whole-brain spectral slope average and BMI (p <1.2e-04). Linear regression model (*fitlm* function in MATLAB 2023b) results including head motion (HM), sex, age, and BMI as independent variables, and whole-brain spectral slope average as a dependent variable. See Figure S3 (left panel).

|  | Estimate | Standard Error | tStat | p value |
| --- | --- | --- | --- | --- |
| (Intercept) | 29.76988 | 1.314382 | 22.64934 | 1.26E-73 |
| HM | -4.95133 | 2.145645 | -2.30762 | 0.021531 |
| sex | -2.39972 | 0.418064 | -5.74007 | 1.88E-08 |
| age | -0.05622 | 0.011488 | -4.89347 | 1.44E-06 |
| BMI | -0.19184 | 0.049291 | -3.892 | 0.000116 |

**Supplementary Table 3.** Relation between spectral slope youthful index (SSYI) and BMI (p < 0.03). Linear regression model (*fitlm* function in MATLAB 2023b) results including head motion (HM), sex, age, and BMI as independent variables, and SSYI as a dependent variable. See Figure S3 (right panel).

|  | Estimate | Standard Error | tStat | p value |
| --- | --- | --- | --- | --- |
| (Intercept) | 0.965069 | 0.070782 | 13.63442 | 5.12E-35 |
| HM | -0.0339 | 0.115547 | -0.29335 | 0.769408 |
| sex | -0.05097 | 0.022514 | -2.26402 | 0.024109 |
| age | -0.00364 | 0.000619 | -5.87831 | 8.76E-09 |
| BMI | -0.00611 | 0.002654 | -2.30175 | 0.021863 |

**
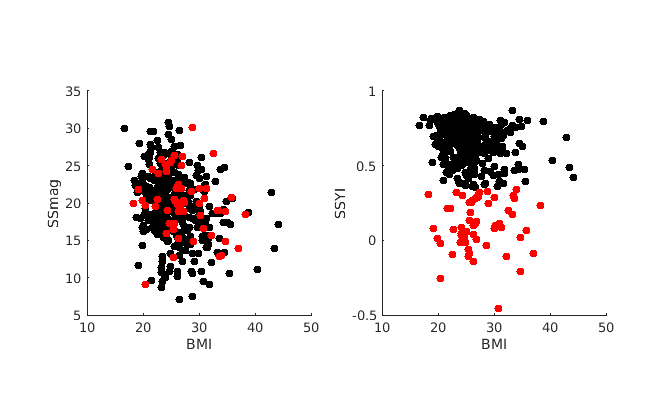
**

**Figure S3.** Relation between BMI and whole-brain spectral slope average (SSmag) and SSYI. Outliers and non-outliers, as shown in Figures 4 and 5 of the main text, are represented by red and black dots, respectively. Spectral slope measures correlate BMI. This relation is statistically significant after accounting for covariates including age, sex, and head motion (Tables S2 and 3).

**T2w frontal lobe white matter intensities are correlated with body mass index (BMI)**

Given that white matter abnormalities are associated with hypertension (Dufouil, de Kersaint-Gilly et al. 2001), we asked whether cardiovascular measures of subjects were related to T2w frontal lobe white matter intensities. We utilized the physiological data provided by CamCAN, which includes systolic blood pressure, diastolic blood pressure, weight, and height. Pulse pressure is calculated as the difference between systolic and diastolic blood pressure. We computed body mass index (BMI) as weight (kg) divided by the square of height (m^2^). BMI is a crude measure of body fat percentage and despite questions regarding the accuracy of BMI as an indicator of cardiovascular health, it is often considered a risk factor for cardiovascular disease (Brown, Higgins et al. 2000). Our findings suggest a significant positive correlation between T2w FLWM intensities and BMI (r = 0.354, p < 1e-06; **Fig. S4**), but no such correlation with pulse pressure.


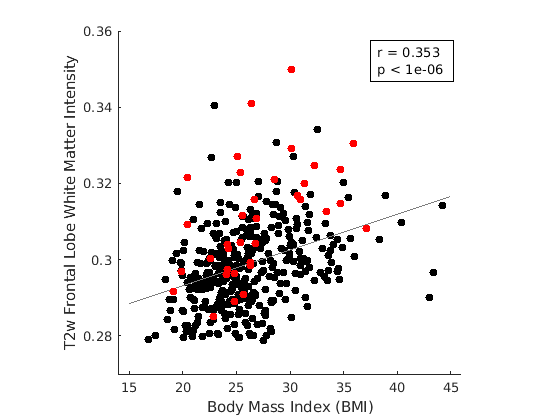


**Figure S4**. Scatterplot demonstrating the relationship between body mass index (BMI) and T2-weighted (T2w) frontal lobe white matter (FLWM) intensity. Outliers and non-outliers, as shown in Figures 4 and 5 of the main text, are represented by red and black dots, respectively. Note the positive correlation between BMI and T2w FLWM intensity.

How does Global Signal Regression affect Spectral Slope?

Global Signal Regression (GSR) zero-centers the distribution of brain-wide correlations and approximately equalizes the prevalence of positive and negative correlations over the whole brain (Fox, Zhang et al. 2009). Although GSR has been controversial in the past (Murphy and Fox 2017), It is now generally accepted that GSR enhances the specificity of resting state network mapping. Thus, GSR is a component of optimal resting state fMRI preprocessing when the objective mapping of resting state networks (Ciric, Rosen et al. 2018, Luppi, Gellersen et al. 2024). However, if the objective is studying the manifestations of fluctuating arousal, then GSR is disadvantageous as arousal is reflected globally in the BOLD signal (Liu, Nalci et al. 2017, Uddin 2020). Whether GSR alters the spectral characteristics of BOLD signals is a pertinent question.

To investigate this question, we evaluated the consistency of spectral slope with and without GSR. The figure below shows power spectra of grey matter (GM) BOLD signals (computed with and without GSR) and the spectrum of the GS in 15 exemplar subjects. As expected, spectra computed without GSR exhibit higher amplitude. The global signal itself shows a steeper spectral slope, suggesting the presence of supra-infraslow activity—an interesting observation that warrants investigation in future work. Importantly, the spectral slope remains largely consistent with or without GSR. This is demonstrated in the rightmost scatter plot of gray matter spectral slope with vs. without GSR. The implication of this finding is that our principle result does not depend on GSR.


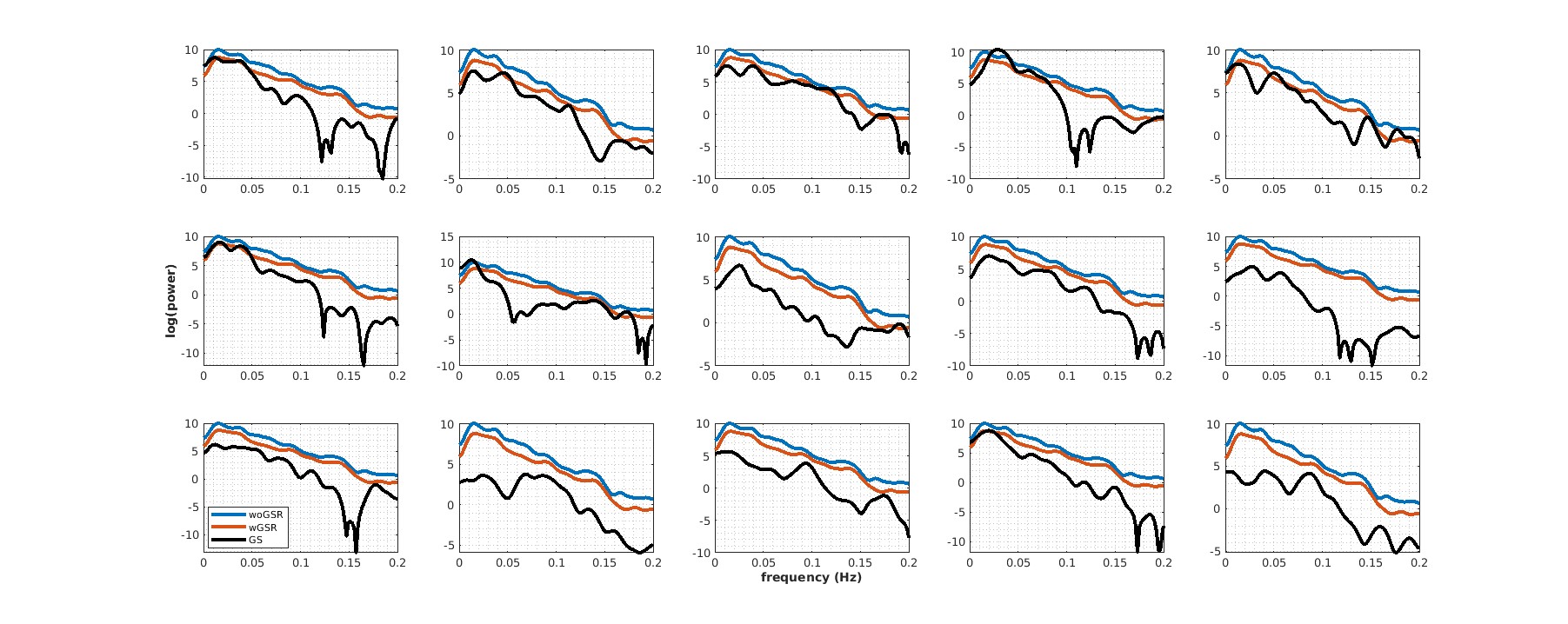


**Figure S5.** Power spectra of grey matter (GM) BOLD signals and global signal in 15 exemplar subjects. Each panel shows one subject’s mean GM power spectrum with GSR (red), without GSR (blue), and the spectrum of global signal (black).


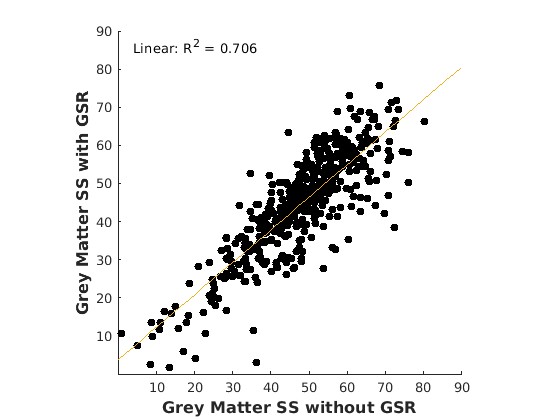


**Figure S6.** Grey matter spectral slope (SS) with and without GSR. Each point represents a single subject. The strong positive correlation suggests that individuals with higher SS in the GSR pipeline also exhibit higher SS in the non-GSR pipeline.

**FC and network dedifferentiation with aging**

Age-related changes in FC and network dedifferentiation have been widely reported (Tomasi and Volkow 2012, Geerligs, Renken et al. 2015, Damoiseaux 2017). However, as shown by (Brier, Thomas et al.), many prior studies did not account for undiagnosed preclinical Alzheimer’s disease. Brier and colleagues excluded subjects with evidence of preclinical AD and found that age-related FC effects are relatively small. Follow-up work (Strain, Brier et al. 2022) confirmed this result, showing that, although healthy aging is associated with reduced global rs-fMRI covariance FC, conventional correlation FC changes are comparatively small. These findings are consistent with earlier reports of multifocal reductions in BOLD signal variability with age (Garrett, McIntosh et al. 2011, Grady and Garrett 2014). Thus, the effects of aging should be studied in datasets free of preclinical pathology using metrics sensitive to BOLD fluctuation amplitude. Accordingly, we evaluate spectral slope, which quantitates the relative contribution of slow vs. fast fluctuations, of subjects free of preclinical pathology in Cam-CAN dataset. Additionally, we note that, while graph-theoretical measures such as degree centrality have been proposed as markers of network dedifferentiation, such measures are highly sensitive to methodological choices, including parcellation scheme, granularity, FC thresholding, as well as quantity of acquired data (Gordon, Laumann et al. 2017, Park, Shimony et al. 2024).

Aiello, M., E. Salvatore, A. Cachia, S. Pappatà, C. Cavaliere, A. Prinster, E. Nicolai, M. Salvatore, J.-C. Baron and M. Quarantelli (2015). "Relationship between simultaneously acquired resting-state regional cerebral glucose metabolism and functional MRI: A PET/MR hybrid scanner study." NeuroImage **113**: 111-121.

Alexander-Bloch, A. F., H. Shou, S. Liu, T. D. Satterthwaite, D. C. Glahn, R. T. Shinohara, S. N. Vandekar and A. Raznahan (2018). "On testing for spatial correspondence between maps of human brain structure and function." Neuroimage **178**: 540-551.

Andrews-Hanna, J. R., A. Z. Snyder, J. L. Vincent, C. Lustig, D. Head, M. E. Raichle and R. L. Buckner (2007). "Disruption of large-scale brain systems in advanced aging." Neuron **56**(5): 924-935.

Baria, A. T., A. Mansour, L. Huang, M. N. Baliki, G. A. Cecchi, M. M. Mesulam and A. V. Apkarian (2013). "Linking human brain local activity fluctuations to structural and functional network architectures." NeuroImage **73**: 144-155.

Bartzokis, G., J. L. Cummings, D. Sultzer, V. W. Henderson, K. H. Nuechterlein and J. Mintz (2003). "White matter structural integrity in healthy aging adults and patients with Alzheimer disease: a magnetic resonance imaging study." Arch Neurol **60**(3): 393-398.

Bates, E., S. M. Wilson, A. P. Saygin, F. Dick, M. I. Sereno, R. T. Knight and N. F. Dronkers (2003). "Voxel-based lesion-symptom mapping." Nat Neurosci **6**(5): 448-450.

Baum, G. L., J. C. Flournoy, M. F. Glasser, M. P. Harms, P. Mair, A. F. P. Sanders, D. M. Barch, R. L. Buckner, S. Bookheimer, M. Dapretto, S. Smith, K. M. Thomas, E. Yacoub, D. C. Van Essen and L. H. Somerville (2022). "Graded Variation in T1w/T2w Ratio during Adolescence: Measurement, Caveats, and Implications for Development of Cortical Myelin." The Journal of Neuroscience: The Official Journal of the Society for Neuroscience **42**(29): 5681-5694.

Beckmann, C. F., M. DeLuca, J. T. Devlin and S. M. Smith (2005). "Investigations into resting-state connectivity using independent component analysis." Philosophical Transactions of the Royal Society of London. Series B, Biological Sciences **360**(1457): 1001-1013.

Bero, A. W., A. Q. Bauer, F. R. Stewart, B. R. White, J. R. Cirrito, M. E. Raichle, J. P. Culver and D. M. Holtzman (2012). "Bidirectional relationship between functional connectivity and amyloid-beta deposition in mouse brain." J Neurosci **32**(13): 4334-4340.

Bero, A. W., P. Yan, J. H. Roh, J. R. Cirrito, F. R. Stewart, M. E. Raichle, J. M. Lee and D. M. Holtzman (2011). "Neuronal activity regulates the regional vulnerability to amyloid-beta deposition." Nat Neurosci **14**(6): 750-756.

Biswal, B., F. Z. Yetkin, V. M. Haughton and J. S. Hyde (1995). "Functional connectivity in the motor cortex of resting human brain using echo-planar MRI." Magnetic Resonance in Medicine **34**(4): 537-541.

Boynton, G. M., S. A. Engel, G. H. Glover and D. J. Heeger (1996). "Linear systems analysis of functional magnetic resonance imaging in human V1." J Neurosci **16**(13): 4207-4221.

Brier, M. R., A. Z. Snyder, A. Tanenbaum, R. A. Rudick, E. Fisher, S. Jones, J. S. Shimony, A. H. Cross, T. L. S. Benzinger and R. T. Naismith (2021). "Quantitative signal properties from standardized MRIs correlate with multiple sclerosis disability." Ann Clin Transl Neurol **8**(5): 1096-1109.

Brier, M. R., J. B. Thomas, A. Z. Snyder, L. Wang, A. M. Fagan, T. Benzinger, J. C. Morris and B. M. Ances (2014). "Unrecognized preclinical Alzheimer disease confounds rs-fcMRI studies of normal aging." Neurology **83**(18): 1613-1619.

Brown, C. D., M. Higgins, K. A. Donato, F. C. Rohde, R. Garrison, E. Obarzanek, N. D. Ernst and M. Horan (2000). "Body mass index and the prevalence of hypertension and dyslipidemia." Obes Res **8**(9): 605-619.

Buckner, R. L., J. Sepulcre, T. Talukdar, F. M. Krienen, H. Liu, T. Hedden, J. R. Andrews-Hanna, R. A. Sperling and K. A. Johnson (2009). "Cortical hubs revealed by intrinsic functional connectivity: mapping, assessment of stability, and relation to Alzheimer's disease." J Neurosci **29**(6): 1860-1873.

Bullmore, E., C. Long, J. Suckling, J. Fadili, G. Calvert, F. Zelaya, T. A. Carpenter and M. Brammer (2001). "Colored noise and computational inference in neurophysiological (fMRI) time series analysis: resampling methods in time and wavelet domains." Hum Brain Mapp **12**(2): 61-78.

Chan, M. K., M. O. Krebs, D. Cox, P. C. Guest, R. H. Yolken, H. Rahmoune, M. Rothermundt, J. Steiner, F. M. Leweke, N. J. van Beveren, D. W. Niebuhr, N. S. Weber, D. N. Cowan, P. Suarez-Pinilla, B. Crespo-Facorro, C. Mam-Lam-Fook, J. Bourgin, R. J. Wenstrup, R. R. Kaldate, J. D. Cooper and S. Bahn (2015). "Development of a blood-based molecular biomarker test for identification of schizophrenia before disease onset." Transl Psychiatry **5**(7): e601.

Ciric, R., A. F. G. Rosen, G. Erus, M. Cieslak, A. Adebimpe, P. A. Cook, D. S. Bassett, C. Davatzikos, D. H. Wolf and T. D. Satterthwaite (2018). "Mitigating head motion artifact in functional connectivity MRI." Nat Protoc **13**(12): 2801-2826.

Cordes, D., V. M. Haughton, K. Arfanakis, G. J. Wendt, P. A. Turski, C. H. Moritz, M. A. Quigley and M. E. Meyerand (2000). "Mapping functionally related regions of brain with functional connectivity MR imaging." AJNR. American journal of neuroradiology **21**(9): 1636-1644.

Damoiseaux, J. S. (2017). "Effects of aging on functional and structural brain connectivity." Neuroimage **160**: 32-40.

Dastur, D. K. (1985). "Cerebral blood flow and metabolism in normal human aging, pathological aging, and senile dementia." J Cereb Blood Flow Metab **5**(1): 1-9.

Deng, S., C. G. Franklin, M. O'Boyle, W. Zhang, B. L. Heyl, P. A. Jerabek, H. Lu and P. T. Fox (2022). "Hemodynamic and metabolic correspondence of resting-state voxel-based physiological metrics in healthy adults." NeuroImage **250**: 118923.

Dickstein, D. L., D. Kabaso, A. B. Rocher, J. I. Luebke, S. L. Wearne and P. R. Hof (2007). "Changes in the structural complexity of the aged brain." Aging cell **6**(3): 275-284.

Dosenbach, N. U., D. A. Fair, A. L. Cohen, B. L. Schlaggar and S. E. Petersen (2008). "A dual-networks architecture of top-down control." Trends Cogn Sci **12**(3): 99-105.

Dosenbach, N. U., D. A. Fair, F. M. Miezin, A. L. Cohen, K. K. Wenger, R. A. Dosenbach, M. D. Fox, A. Z. Snyder, J. L. Vincent, M. E. Raichle, B. L. Schlaggar and S. E. Petersen (2007). "Distinct brain networks for adaptive and stable task control in humans." Proc Natl Acad Sci U S A **104**(26): 11073-11078.

Dosenbach, N. U. F., M. Raichle and E. M. Gordon (2024). The brain’s cingulo-opercular action-mode network, OSF.

Doucet, G., M. Naveau, L. Petit, N. Delcroix, L. Zago, F. Crivello, G. Jobard, N. Tzourio-Mazoyer, B. Mazoyer, E. Mellet and M. Joliot (2011). "Brain activity at rest: a multiscale hierarchical functional organization." J Neurophysiol **105**(6): 2753-2763.

Dufouil, C., A. de Kersaint-Gilly, V. Besancon, C. Levy, E. Auffray, L. Brunnereau, A. Alperovitch and C. Tzourio (2001). "Longitudinal study of blood pressure and white matter hyperintensities: the EVA MRI Cohort." Neurology **56**(7): 921-926.

Dworetsky, A., B. A. Seitzman, B. Adeyemo, M. Neta, R. S. Coalson, S. E. Petersen and C. Gratton (2021). "Probabilistic mapping of human functional brain networks identifies regions of high group consensus." Neuroimage **237**: 118164.

Fagan, A. M., C. Xiong, M. S. Jasielec, R. J. Bateman, A. M. Goate, T. L. Benzinger, B. Ghetti, R. N. Martins, C. L. Masters, R. Mayeux, J. M. Ringman, M. N. Rossor, S. Salloway, P. R. Schofield, R. A. Sperling, D. Marcus, N. J. Cairns, V. D. Buckles, J. H. Ladenson, J. C. Morris, D. M. Holtzman and N. Dominantly Inherited Alzheimer (2014). "Longitudinal change in CSF biomarkers in autosomal-dominant Alzheimer's disease." Sci Transl Med **6**(226): 226ra230.

Fox, M. D., M. Corbetta, A. Z. Snyder, J. L. Vincent and M. E. Raichle (2006). "Spontaneous neuronal activity distinguishes human dorsal and ventral attention systems." Proceedings of the National Academy of Sciences **103**(26): 10046-10051.

Fox, M. D., D. Zhang, A. Z. Snyder and M. E. Raichle (2009). "The global signal and observed anticorrelated resting state brain networks." J Neurophysiol **101**(6): 3270-3283.

Garrett, D. D., N. Kovacevic, A. R. McIntosh and C. L. Grady (2013). "The modulation of BOLD variability between cognitive states varies by age and processing speed." Cereb Cortex **23**(3): 684-693.

Garrett, D. D., A. R. McIntosh and C. L. Grady (2011). "Moment-to-moment signal variability in the human brain can inform models of stochastic facilitation now." Nat Rev Neurosci **12**(10): 612; author reply 612.

Geerligs, L., R. J. Renken, E. Saliasi, N. M. Maurits and M. M. Lorist (2015). "A Brain-Wide Study of Age-Related Changes in Functional Connectivity." Cereb Cortex **25**(7): 1987-1999.

Goldman, M. S., A. Compte and X. J. Wang (2009). Neural Integrator Models. Encyclopedia of Neuroscience, Elsevier Ltd**:** 165-178.

Gorbunova, V., A. Seluanov, Z. Mao and C. Hine (2007). "Changes in DNA repair during aging." Nucleic Acids Res **35**(22): 7466-7474.

Gordon, E. M., T. O. Laumann, B. Adeyemo, J. F. Huckins, W. M. Kelley and S. E. Petersen (2016). "Generation and Evaluation of a Cortical Area Parcellation from Resting-State Correlations." Cereb Cortex **26**(1): 288-303.

Gordon, E. M., T. O. Laumann, A. W. Gilmore, D. J. Newbold, D. J. Greene, J. J. Berg, M. Ortega, C. Hoyt-Drazen, C. Gratton, H. Sun, J. M. Hampton, R. S. Coalson, A. L. Nguyen, K. B. McDermott, J. S. Shimony, A. Z. Snyder, B. L. Schlaggar, S. E. Petersen, S. M. Nelson and N. U. F. Dosenbach (2017). "Precision Functional Mapping of Individual Human Brains." Neuron **95**(4): 791-807 e797.

Gotts, S. J., A. W. Gilmore and A. Martin (2020). "Brain networks, dimensionality, and global signal averaging in resting-state fMRI: Hierarchical network structure results in low-dimensional spatiotemporal dynamics." Neuroimage **205**: 116289.

Goyal, M. S., T. Blazey, N. V. Metcalf, M. P. McAvoy, J. F. Strain, M. Rahmani, T. J. Durbin, C. Xiong, T. L. Benzinger, J. C. Morris, M. E. Raichle and A. G. Vlassenko (2023). "Brain aerobic glycolysis and resilience in Alzheimer disease." Proc Natl Acad Sci U S A **120**(7): e2212256120.

Goyal, M. S., T. M. Blazey, Y. Su, L. E. Couture, T. J. Durbin, R. J. Bateman, T. L. Benzinger, J. C. Morris, M. E. Raichle and A. G. Vlassenko (2019). "Persistent metabolic youth in the aging female brain." Proc Natl Acad Sci U S A **116**(8): 3251-3255.

Goyal, M. S., M. Hawrylycz, J. A. Miller, A. Z. Snyder and M. E. Raichle (2014). "Aerobic Glycolysis in the Human Brain Is Associated with Development and Neotenous Gene Expression." Cell Metabolism **19**(1): 49-57.

Goyal, M. S., A. G. Vlassenko, T. M. Blazey, Y. Su, L. E. Couture, T. J. Durbin, R. J. Bateman, T. L. Benzinger, J. C. Morris and M. E. Raichle (2017). "Loss of Brain Aerobic Glycolysis in Normal Human Aging." Cell Metab **26**(2): 353-360 e353.

Grady, C. L. and D. D. Garrett (2014). "Understanding variability in the BOLD signal and why it matters for aging." Brain Imaging and Behavior **8**(2): 274-283.

Greicius, M. D., B. Krasnow, A. L. Reiss and V. Menon (2003). "Functional connectivity in the resting brain: a network analysis of the default mode hypothesis." Proceedings of the National Academy of Sciences of the United States of America **100**(1): 253-258.

Hampson, M., B. S. Peterson, P. Skudlarski, J. C. Gatenby and J. C. Gore (2002). "Detection of functional connectivity using temporal correlations in MR images." Human Brain Mapping **15**(4): 247-262.

He, B. J. (2011). "Scale-Free Properties of the Functional Magnetic Resonance Imaging Signal during Rest and Task." Journal of Neuroscience **31**(39): 13786-13795.

Kety, S. S. (1956). "Human cerebral blood flow and oxygen consumption as related to aging." J Chronic Dis **3**(5): 478-486.

Kirkwood, T. B. (2005). "Understanding the odd science of aging." Cell **120**(4): 437-447.

Kuhl, D. E., E. J. Metter, W. H. Riege and M. E. Phelps (1982). "Effects of human aging on patterns of local cerebral glucose utilization determined by the [18F]fluorodeoxyglucose method." J Cereb Blood Flow Metab **2**(2): 163-171.

Kuipers, J. B. (1999). Quaternions and rotation sequences : a primer with applications to orbits, aerospace, and virtual reality. Princeton, N.J., Princeton University Press.

Laumann, T. O. and A. Z. Snyder (2021). "Brain activity is not only for thinking." Current Opinion in Behavioral Sciences **40**: 130-136.

Leech, R. and D. J. Sharp (2014). "The role of the posterior cingulate cortex in cognition and disease." Brain **137**(Pt 1): 12-32.

Liakakis, G., J. Nickel and R. J. Seitz (2011). "Diversity of the inferior frontal gyrus--a meta-analysis of neuroimaging studies." Behav Brain Res **225**(1): 341-347.

Liu, H., Y. Yang, Y. Xia, W. Zhu, R. K. Leak, Z. Wei, J. Wang and X. Hu (2017). "Aging of cerebral white matter." Ageing Res Rev **34**: 64-76.

Liu, T. T. (2016). "Noise contributions to the fMRI signal: An overview." Neuroimage **143**: 141-151.

Liu, T. T., A. Nalci and M. Falahpour (2017). "The global signal in fMRI: Nuisance or Information?" Neuroimage **150**: 213-229.

Lombard, D. B., K. F. Chua, R. Mostoslavsky, S. Franco, M. Gostissa and F. W. Alt (2005). "DNA repair, genome stability, and aging." Cell **120**(4): 497-512.

Lopez-Otin, C., M. A. Blasco, L. Partridge, M. Serrano and G. Kroemer (2013). "The hallmarks of aging." Cell **153**(6): 1194-1217.

Lowe, M. J., B. J. Mock and J. A. Sorenson (1998). "Functional connectivity in single and multislice echoplanar imaging using resting-state fluctuations." NeuroImage **7**(2): 119-132.

Luckett, P. H., J. J. Lee, K. Y. Park, R. V. Raut, K. L. Meeker, E. M. Gordon, A. Z. Snyder, B. M. Ances, E. C. Leuthardt and J. S. Shimony (2022). "Resting state network mapping in individuals using deep learning." Front Neurol **13**: 1055437.

Luo, J., F. Agboola, E. Grant, C. L. Masters, M. S. Albert, S. C. Johnson, E. M. McDade, J. Voglein, A. M. Fagan, T. Benzinger, P. Massoumzadeh, J. Hassenstab, R. J. Bateman, J. C. Morris, R. J. Perrin, J. Chhatwal, M. Jucker, B. Ghetti, C. Cruchaga, N. R. Graff-Radford, P. R. Schofield, H. Mori and C. Xiong (2020). "Sequence of Alzheimer disease biomarker changes in cognitively normal adults: A cross-sectional study." Neurology **95**(23): e3104-e3116.

Luo, J., Y. Ma, F. J. Agboola, E. Grant, J. C. Morris, E. McDade, A. M. Fagan, T. L. S. Benzinger, J. Hassenstab, R. J. Bateman, R. J. Perrin, B. A. Gordon, M. Goyal, J. F. Strain, I. Yakushev, G. S. Day, C. Xiong and N. for Dominantly Inherited Alzheimer (2023). "Longitudinal Relationships of White Matter Hyperintensities and Alzheimer Disease Biomarkers Across the Adult Life Span." Neurology **101**(2): e164-e177.

Luppi, A. I., H. M. Gellersen, Z. Q. Liu, A. R. D. Peattie, A. E. Manktelow, R. Adapa, A. M. Owen, L. Naci, D. K. Menon, S. I. Dimitriadis and E. A. Stamatakis (2024). "Systematic evaluation of fMRI data-processing pipelines for consistent functional connectomics." Nat Commun **15**(1): 4745.

Mitra, A. and M. E. Raichle (2016). "How networks communicate: propagation patterns in spontaneous brain activity." Philos Trans R Soc Lond B Biol Sci **371**(1705).

Murphy, K. and M. D. Fox (2017). "Towards a consensus regarding global signal regression for resting state functional connectivity MRI." Neuroimage **154**: 169-173.

Nachev, P., C. Kennard and M. Husain (2008). "Functional role of the supplementary and pre-supplementary motor areas." Nat Rev Neurosci **9**(11): 856-869.

Nugent, A. C., A. Martinez, A. D'Alfonso, C. A. Zarate and W. H. Theodore (2015). "The Relationship between Glucose Metabolism, Resting-State fMRI BOLD Signal, and GABAA-Binding Potential: A Preliminary Study in Healthy Subjects and Those with Temporal Lobe Epilepsy." Journal of Cerebral Blood Flow & Metabolism **35**(4): 583-591.

Ojemann, J. G., E. Akbudak, A. Z. Snyder, R. C. McKinstry, M. E. Raichle and T. E. Conturo (1997). "Anatomic localization and quantitative analysis of gradient refocused echo-planar fMRI susceptibility artifacts." Neuroimage **6**(3): 156-167.

Palva, J. M. and S. Palva (2012). "Infra-slow fluctuations in electrophysiological recordings, blood-oxygenation-level-dependent signals, and psychophysical time series." Neuroimage **62**(4): 2201-2211.

Park, K. Y., J. S. Shimony, S. Chakrabarty, A. B. Tanenbaum, C. D. Hacker, K. M. Donovan, P. H. Luckett, M. Milchenko, A. Sotiras, D. S. Marcus, E. C. Leuthardt and A. Z. Snyder (2024). "Optimal approaches to analyzing functional MRI data in glioma patients." J Neurosci Methods **402**: 110011.

Park, K. Y., A. Z. Snyder, M. Olufawo, G. Trevino, P. H. Luckett, B. Lamichhane, T. Xie, J. J. Lee, J. S. Shimony and E. C. Leuthardt (2023). "Glioblastoma induces whole-brain spectral change in resting state fMRI: Associations with clinical comorbidities and overall survival." NeuroImage: Clinical **39**: 103476.

Pereira, J. B., P. Svenningsson, D. Weintraub, K. Bronnick, A. Lebedev, E. Westman and D. Aarsland (2014). "Initial cognitive decline is associated with cortical thinning in early Parkinson disease." Neurology **82**(22): 2017-2025.

Power, J. D., K. A. Barnes, A. Z. Snyder, B. L. Schlaggar and S. E. Petersen (2012). "Spurious but systematic correlations in functional connectivity MRI networks arise from subject motion." Neuroimage **59**(3): 2142-2154.

Raichle, M. E. and M. A. Mintun (2006). "Brain work and brain imaging." Annual Review of Neuroscience **29**: 449-476.

Rashid, T., K. Li, J. B. Toledo, I. Nasrallah, N. M. Pajewski, S. Dolui, J. Detre, D. A. Wolk, H. Liu, S. R. Heckbert, R. N. Bryan, J. Williamson, C. Davatzikos, S. Seshadri, L. J. Launer and M. Habes (2023). "Association of Intensive vs Standard Blood Pressure Control With Regional Changes in Cerebral Small Vessel Disease Biomarkers: Post Hoc Secondary Analysis of the SPRINT MIND Randomized Clinical Trial." JAMA Netw Open **6**(3): e231055.

Raut, R. V., A. Z. Snyder and M. E. Raichle (2020). "Hierarchical dynamics as a macroscopic organizing principle of the human brain." Proc Natl Acad Sci U S A **117**(34): 20890-20897.

Rousseeuw, P. J. (1987). "Silhouettes: A graphical aid to the interpretation and validation of cluster analysis." Journal of Computational and Applied Mathematics **20**: 53-65.

Sala-Llonch, R., D. Bartres-Faz and C. Junque (2015). "Reorganization of brain networks in aging: a review of functional connectivity studies." Front Psychol **6**: 663.

Salat, D. H., J. A. Kaye and J. S. Janowsky (1999). "Prefrontal gray and white matter volumes in healthy aging and Alzheimer disease." Arch Neurol **56**(3): 338-344.

Sattarivand, M., M. Kusano, I. Poon and C. Caldwell (2012). "Symmetric geometric transfer matrix partial volume correction for PET imaging: principle, validation and robustness." Phys Med Biol **57**(21): 7101-7116.

Schaefer, A., R. Kong, E. M. Gordon, T. O. Laumann, X. N. Zuo, A. J. Holmes, S. B. Eickhoff and B. T. T. Yeo (2018). "Local-Global Parcellation of the Human Cerebral Cortex from Intrinsic Functional Connectivity MRI." Cereb Cortex **28**(9): 3095-3114.

Seeley, W. W., V. Menon, A. F. Schatzberg, J. Keller, G. H. Glover, H. Kenna, A. L. Reiss and M. D. Greicius (2007). "Dissociable Intrinsic Connectivity Networks for Salience Processing and Executive Control." Journal of Neuroscience **27**(9): 2349-2356.

Seitzman, B. A., C. Gratton, S. Marek, R. V. Raut, N. U. F. Dosenbach, B. L. Schlaggar, S. E. Petersen and D. J. Greene (2020). "A set of functionally-defined brain regions with improved representation of the subcortex and cerebellum." Neuroimage **206**: 116290.

Shafto, M. A., L. K. Tyler, M. Dixon, J. R. Taylor, J. B. Rowe, R. Cusack, A. J. Calder, W. D. Marslen-Wilson, J. Duncan, T. Dalgleish, R. N. Henson, C. Brayne, F. E. Matthews and Cam-CAN (2014). "The Cambridge Centre for Ageing and Neuroscience (Cam-CAN) study protocol: a cross-sectional, lifespan, multidisciplinary examination of healthy cognitive ageing." BMC Neurology **14**(1): 204.

Shulman, G. L., D. L. Pope, S. V. Astafiev, M. P. McAvoy, A. Z. Snyder and M. Corbetta (2010). "Right hemisphere dominance during spatial selective attention and target detection occurs outside the dorsal frontoparietal network." J Neurosci **30**(10): 3640-3651.

Strain, J. F., M. R. Brier, A. Tanenbaum, B. A. Gordon, J. E. McCarthy, A. Dincer, D. S. Marcus, J. P. Chhatwal, N. R. Graff-Radford, G. S. Day, C. la Fougere, R. J. Perrin, S. Salloway, P. R. Schofield, I. Yakushev, T. Ikeuchi, J. Voglein, J. C. Morris, T. L. S. Benzinger, R. J. Bateman, B. M. Ances, A. Z. Snyder and N. Dominantly Inherited Alzheimer (2022). "Covariance-based vs. correlation-based functional connectivity dissociates healthy aging from Alzheimer disease." Neuroimage **261**: 119511.

Sydnor, V. J., B. Larsen, J. Seidlitz, A. Adebimpe, A. F. Alexander-Bloch, D. S. Bassett, M. A. Bertolero, M. Cieslak, S. Covitz, Y. Fan, R. E. Gur, R. C. Gur, A. P. Mackey, T. M. Moore, D. R. Roalf, R. T. Shinohara and T. D. Satterthwaite (2023). "Intrinsic activity development unfolds along a sensorimotor–association cortical axis in youth." Nature Neuroscience **26**(4): 638-649.

Tagliazucchi, E., F. von Wegner, A. Morzelewski, V. Brodbeck, K. Jahnke and H. Laufs (2013). "Breakdown of long-range temporal dependence in default mode and attention networks during deep sleep." Proceedings of the National Academy of Sciences **110**(38): 15419-15424.

Taylor, J. R., N. Williams, R. Cusack, T. Auer, M. A. Shafto, M. Dixon, L. K. Tyler, Cam-CAN and R. N. Henson (2017). "The Cambridge Centre for Ageing and Neuroscience (Cam-CAN) data repository: Structural and functional MRI, MEG, and cognitive data from a cross-sectional adult lifespan sample." NeuroImage **144**: 262-269.

Tomasi, D. and N. D. Volkow (2012). "Aging and functional brain networks." Mol Psychiatry **17**(5): 471, 549-458.

Tononi, G. and C. Cirelli (2014). "Sleep and the price of plasticity: from synaptic and cellular homeostasis to memory consolidation and integration." Neuron **81**(1): 12-34.

Uddin, L. Q. (2020). "Bring the Noise: Reconceptualizing Spontaneous Neural Activity." Trends Cogn Sci **24**(9): 734-746.

Van Essen, D. C., K. Ugurbil, E. Auerbach, D. Barch, T. E. Behrens, R. Bucholz, A. Chang, L. Chen, M. Corbetta, S. W. Curtiss, S. Della Penna, D. Feinberg, M. F. Glasser, N. Harel, A. C. Heath, L. Larson-Prior, D. Marcus, G. Michalareas, S. Moeller, R. Oostenveld, S. E. Petersen, F. Prior, B. L. Schlaggar, S. M. Smith, A. Z. Snyder, J. Xu, E. Yacoub and W. U.-M. H. Consortium (2012). "The Human Connectome Project: a data acquisition perspective." Neuroimage **62**(4): 2222-2231.

Walhovd, K. B., A. B. Storsve, L. T. Westlye, C. A. Drevon and A. M. Fjell (2014). "Blood markers of fatty acids and vitamin D, cardiovascular measures, body mass index, and physical activity relate to longitudinal cortical thinning in normal aging." Neurobiol Aging **35**(5): 1055-1064.

Wardlaw, J. M., C. Smith and M. Dichgans (2013). "Mechanisms of sporadic cerebral small vessel disease: insights from neuroimaging." Lancet Neurol **12**(5): 483-497.

Wu, K. and L. L. Gollo (2025). "Mapping and modeling age-related changes in intrinsic neural timescales." Commun Biol **8**(1): 167.

Zang, Y.-F., Y. He, C.-Z. Zhu, Q.-J. Cao, M.-Q. Sui, M. Liang, L.-X. Tian, T.-Z. Jiang and Y.-F. Wang (2007). "Altered baseline brain activity in children with ADHD revealed by resting-state functional MRI." Brain & Development **29**(2): 83-91.

Zou, Q.-H., C.-Z. Zhu, Y. Yang, X.-N. Zuo, X.-Y. Long, Q.-J. Cao, Y.-F. Wang and Y.-F. Zang (2008). "An improved approach to detection of amplitude of low-frequency fluctuation (ALFF) for resting-state fMRI: Fractional ALFF." Journal of Neuroscience Methods **172**(1): 137-141.
